# Female sex and femininity independently associate with common somatic symptom trajectories

**DOI:** 10.1017/S0033291720004043

**Published:** 2022-08

**Authors:** Aranka V. Ballering, Klaas J. Wardenaar, Tim C. olde Hartman, Judith G. M. Rosmalen

**Affiliations:** 1 University of Groningen, University Medical Center of Groningen, Interdisciplinary Center Psychopathology and Emotion Regulation (ICPE), P.O. Box 30.001, 9700 RB, Groningen, The Netherlands; 2 Department of Primary and Community Care, Radboud University Medical Center, P.O. Box 9101, 6500 HB, Nijmegen, The Netherlands

**Keywords:** Common somatic symptoms, gender differences, latent class trajectory modeling, sex differences

## Abstract

**Background:**

Multiple predictors have been associated with persistent somatic symptoms. However, previous studies problematically defined the persistence of symptoms, conflated participants' sex and gender, and focused on patient populations. Therefore, we studied associations between predictors, especially sex and gender, and longitudinal patterns of somatic symptoms in the general adult population. We also assessed whether predictors for persisting symptoms differ between sexes.

**Method:**

To identify developmental trajectories of somatic symptoms, assessed by the SCL-90 SOM, we used latent class trajectory modeling in the Dutch Lifelines Cohort Study [*N* = 150 494; 58.6% female; median time to follow-up: 46.0 (min–max: 22.0–123.0) months]. To identify predictors of trajectories, we applied multiple logistic regression analyses. Predictors were measured by surveys at baseline and a composite gender index was previously developed.

**Results:**

A five-class linear LCGA model fitted the data best: 93.7% of the population had a stable symptom trajectory, whereas 1.5% and 4.8% of the population had a consistently increasing or decreasing symptom trajectory, respectively. Female sex predicted severe, stable symptom severity (OR 1.74, 95% CI 1.36–2.22), but not increasing symptom severity (OR 1.15, 95% CI 0.99–1.40). Femininity was protective hereof (OR 0.60, 95% CI 0.44–0.82 and OR 0.66, 95% CI 0.51–0.85, respectively). Merely a few predictors of symptom severity, for instance hours of paid employment and physical functioning, differed in strength between sexes. Yet, effect sizes were small.

**Conclusion:**

Female sex and femininity predict symptom trajectories. No large sex differences in the strength of additional predictors were found, thus it may not be clinically useful to distinguish between predictors specific to male or female patients of persistent somatic symptoms.

## Introduction

A substantial proportion of the general practitioner (GP) visits in the Netherlands (13–43%) are related to common somatic symptoms for which no sufficient cause can be found after adequate physical examinations and interventions (Olde Hartman et al., 2013; Verhaak, Meijer, Visser, & Wolters, 2006). Persistence of these somatic symptoms is associated with increased functional impairment, feelings of internalized stigma and social isolation (Dirkzwager & Verhaak, 2007). In addition to personal hardship, persistent common somatic symptoms may pose an economic burden on both an individual and societal level (Joustra, Janssens, Bültmann, & Rosmalen, 2015; Konnopka et al., 2012).

A systematic review from 2009 showed that 10–30% of patients with medically unexplained somatic symptoms attending the GP or secondary care clinic did not improve during their follow-up period of 6–15 months (olde Hartman et al., 2009). More recent studies suggest higher rates of non-remission in primary care with 0.5 year (55.1%) (Lamahewa, Buszewicz, Walters, Marston, & Nazareth, 2019), 1 year (51.2%) (Steinbrecher & Hiller, 2011), and 2 years follow-up (56.8% and 37.1%) (Budtz-Lilly, Vestergaard, Fink, Carlsen, & Rosendal, 2015; Claassen-van Dessel, van der Wouden, Hoekstra, Dekker, & van der Horst, 2018). However, studies describing the persistence of common somatic symptoms are difficult to compare due to methodological differences. Furthermore, these figures might not be representative of the general adult population. The prognosis of common somatic symptoms in the general population is likely to be more favorable, since by definition patient populations suffer from symptoms that they regard serious enough to visit a physician. To the best of our knowledge, only one recent study on the persistence of unexplained common somatic symptoms has been conducted in the adult general population, indicating that 36.4% of people had persistent common somatic symptoms measured over 3 years (van Eck van der Sluijs et al., 2018).

A variety of predictors for persisting common somatic symptoms has been identified in adolescent and adult populations, including female sex (Janssens, Klis, Kingma, Oldehinkel, & Rosmalen, 2014; Steinbrecher & Hiller, 2011), physical and psychiatric comorbidities (olde Hartman et al., 2009), symptom characteristics (such as the duration, severity and heterogeneity) (Budtz-Lilly et al., 2015; Kooiman, Bolk, Rooijmans, & Trijsburg, 2004), and psychological traits (such as neuroticism, perfectionism, and health perceptions) (Bonvanie, Rosmalen, van Rhede van der Kloot, Oldehinkel, & Janssens, 2015; De Gucht, Fischler, & Heiser, 2004; Janssens et al., 2014). Identification of predictors for persisting somatic symptoms is pivotal, as it allows for early detection, diagnosis, and treatment and it could provide concrete starting points for interventions aiming to reduce or prevent such symptoms as well (olde Hartman et al., 2009). However, the definition of the persistence of symptoms in most aforementioned studies aiming to identify predictors was suboptimal. For example, persistence was defined based on an arbitrary number of contacts with the GP (Verhaak et al., 2006). This does not distinguish between one's symptoms and healthcare-seeking behavior, which is especially problematic given the observation that patients who do not return to the GP often still experience symptoms (Koch et al., 2009). Furthermore, it remains unknown whether these predictors differ between women and men.

Recently, a cross-sectional study found that not only female sex, but also feminine gender, which encompasses the roles, behaviors, identities, and relationships of women prescribed by societal norms in a given context (Johnson, Greaves, & Repta, 2009), is associated with the severity of common somatic symptoms and prevalence of chronic diseases (Ballering, Bonvanie, Olde Hartman, Monden, & Rosmalen, 2020). Gender, and its embodiment, is more dynamic than one's biological sex. Yet, gender and sex are often conflated in research. Therefore, to date, it remains unknown whether sex and gender independently impact the severity and persistence of somatic symptoms in the general adult population. In addition, most studies assessing the severity and persistence of somatic symptoms have considered the cohort under study as a homogeneous population, while the longitudinal patterns of symptom development may show significant heterogeneity in their directions (Claassen-van Dessel et al., 2018). This means that in most studies variable patterns of somatic symptoms over time remain undetected.

We present the first large epidemiological cohort study to identify the predictors of longitudinal patterns of somatic symptoms in the general adult population, with a special emphasis on sex and gender differences. First, we will use latent class trajectory modeling to identify developmental patterns of symptom severity. Second, we will assess which predictors are associated with different trajectories. Third, we aim to study whether identified predictors for the persistence of common somatic symptoms differ between females and males. We hypothesize that female sex and femininity associate with increased severity of common somatic symptoms.

## Methods

### Setting

This study is based on data collected within the Dutch Lifelines Cohort Study. The Lifelines Cohort Study is a multi-disciplinary prospective population-based cohort study examining in a unique three-generation design the health and health-related behaviors of 167 729 persons living in the North of The Netherlands. It employs a broad range of investigative procedures in assessing the biomedical, socio-demographic, behavioral, physical, and psychological factors, which contribute to the health and disease of the general population, with a special focus on multi-morbidity and complex genetics. Extensive information on the cohort, design considerations, and recruitment procedures is provided elsewhere (Klijs et al., 2015; Scholtens et al., 2015). The Lifelines Cohort Study is performed according to the principles of the Declaration of Helsinki and in accordance with the UMCG's research code. The Lifelines Cohort Study is approved by the Medical Ethical Committee of the University Medical Center Groningen, The Netherlands (Scholtens et al., 2015). For the current study, we adhered to the STROBE statement and GRoLTS guidelines for reporting of our findings (Van De Schoot, Sijbrandij, Winter, Depaoli, & Vermunt, 2017; von Elm et al., 2014). To conform to the SAGER guidelines we reported our findings stratified by sex (Heidari, Babor, De Castro, Tort, & Curno, 2016).

### Participants

Participants completed questionnaires on multiple topics including, but not limited to, demographics, health, personality, psychological and somatic symptoms, and psychosocial characteristics. These questionnaires asked for participants' biological sex. Hence, we refer to the participants as male and female, whereas we refer to masculinity and femininity when discussing gender.

In the current study, we used data from the adult participants gathered at four time points: at baseline [*n* = 148 643; mean age 44.2 years (s.d. = 12.8); 58.6% females], at a first follow-up time point [*n* = 124 443; mean age 46.5 years (s.d. = 12.8); 59.3% female; median time to follow-up: 13.0 (10–93) months], after a second follow-up time point [*n* = 95 137; mean age 48.1 years (s.d. = 12.8); 59.8% female; median time to follow-up: 25.0 (22–92) months], and after a third follow-up time point [*n* = 90 077; mean age 49.8 years (s.d. = 12.6); 59.1% females; median time to follow-up: 46.0 (22–123) months]. A more detailed overview of the population included is provided in online Supplementary Appendix A. Attrition rates after 1.5, 3, and 4 years were 16.8%, 36.2%, and 39.6%, respectively, compared to baseline. We did not find any indication for relevant systematic attrition: no meaningful associations between potential predictors of the severity of common somatic symptoms and attrition rates were found.

### Variables

We assessed common somatic symptom severity by means of the 12-item ordinal Symptom CheckList-90 Somatization subscale (SCL-90 SOM; online Supplementary Appendix B). The 12 items refer to how much bother or distress someone experienced the past week due to somatic symptoms. This scale has been recommended for large-scale studies and has been shown to have sufficient measurement invariance over time, which makes it suitable to assess the measured concepts repeatedly over time (Rytilä-Manninen et al., 2016; Zijlema et al., 2013). The potential predictors for the persistence of common somatic symptoms, all assessed at baseline, are described in Table 1. Femininity was operationalized via a recently developed gender index (Ballering et al., 2020), which accounts for the time-, place-, and society-bound nature of gender. In a subsample of adult Lifelines participants, with no suspected intersex condition or non-conform gender identity, a LASSO logistic regression model that predicts the participants' sex by means of psychosocial characteristics, including but not limited to hobbies, type of profession, dietary preferences, and time spent on household tasks, was calculated. In total, 85 unique psychosocial variables were included in the model (AUC = 92%) and thus gendered. The included psychosocial predictors cover predominantly gender roles, and therefore reflect the gender roles as adhered to in the Lifelines cohort. The obtained estimates of the regression coefficients were applied to all adult Lifelines participants, providing each participant with an individual score on the gender index, i.e. participants' adherence to the gendered psychosocial variables. The gender index ranges from 0%, equaling masculinity, to 100%, equaling femininity. We assessed the multicollinearity of the predictor variables by means of the variance inflation factor (VIF). We found no indication of problems with multicollinearity, as VIF was <5 in all analyses (Miles & Shevlin, 2001).

**Table 1.** Overview of potential predictors for persistent common somatic symptoms (all assessed at baseline)

Potential predictor	Operationalization of predictor	Minimum–maximum in sample
Age	Municipally-registered age in years	18–92 years
Sex	Municipally-registered sex	Male Female
Gender	Gender Index [for an in-depth discussion of the construction of the gender index we refer to Ballering et al. (2020)]	0% (reflecting masculinity) – 100% (reflecting femininity)
Educational level	Self-reported education (van Zon, Snieder, Bültmann, and Reijneveld, 2017)	High educational level Medium educational level Low educational level
Employment	Self-reported hours of paid work	0–60 h
Chronic somatic diseases	Self-reported lifetime prevalence of chronic somatic diseases. A selection of diseases was included, based on (i) availability of data; (ii) the rankings of the Dutch Ministry of Health, Welfare and Sports of diseases causing the greatest loss of healthy life years in the adult general population; and (iii) previous literature (Dutch Ministry of Health, Welfare and Sports, 2019; Gijsen et al., 2013; Hoeymans, Gijsen, & Slobbe, 2013; van Eck van der Sluijs et al., 2018). Sex-specific diseases (e.g. prostate cancer) were excluded. Chronic somatic diseases included respiratory diseases (COPD, asthma), cardiovascular disorders (stroke, arrhythmia, heart attack, heart failures), intestinal disorders (Crohn's disease, ulcerative colitis, stomach ulcers), diabetes mellitus (type 1 and type 2), arthritis, osteoporosis, migraine, cancer (skin, colon, and lung cancer), dementia, Parkinson's disease, kidney failure, epilepsy, rheumatoid arthritis, and multiple sclerosis	0–10 diseases
Physical functioning	Self-reported physical functioning [the mean score of the 10 items on the physical functioning subscale of the RAND-36 (Aaronson et al., 1998)]	0–100 points
Emotional well-being	Self-reported emotional well-being [the mean score of the five items on the emotional well-being subscale of the RAND-36 (Aaronson et al., 1998)]	0–100 points
Self-rated health	Self-reported health [the mean score of the five items on the general health perception subscale of the RAND-36 (Aaronson et al., 1998)]	0–100 points
Negative affect	Self-reported negative affect [the mean score of the 10 items on the negative affect subscale of the PANAS (Hill, Van Boxtel, Ponds, Houx, & Jolles, 2005)]	0–4 points
Positive affect	Self-reported positive affect [the mean score of the 10 items on the positive affect subscale of the PANAS (Hill et al., 2005)]	0–4 points
Personality traits	Self-reported outcomes of the NEO PI R. Of 8 subscales of the NEO PI R, namely hostility, self-consciousness, impulsiveness, vulnerability, self-discipline, competence, deliberation, and excitement, the mean score was calculated [each subscale consists of 8 items (Costa & McCrae, 1992)]	0–4 points
Negative life events	Self-reported presence of negative life events in the past year, based on the List of Threatening Experiences (Brugha & Cragg, 1990; Rosmalen, Bos, & de Jonge, 2012)	Any threatening experience present No threatening experience present
Long-term difficulties	Self-reported extent of long-term difficulties and stress, either somewhat or very much, based on the Long-term Difficulties Inventory (Rosmalen et al., 2012)	Any long-term difficulty present No long-term difficulty present
Self-reported mood disorders	Self-reported lifetime prevalence of mood disorders (Mood disorders, based on DSM-IV, include major depression and bipolar disorder. No data on dysthymia was available.)	Any mood disorder present No mood disorder present
Self-reported anxiety disorders	Self-reported lifetime prevalence of anxiety disorders (anxiety disorders, based on DSM-IV criteria, include panic disorder, agoraphobia, specific phobia, and generalized anxiety disorder)	Any anxiety disorder present No anxiety disorder present

### Statistical analyses

To identify different developmental trajectories of common somatic symptoms over time, latent class trajectory modeling was conducted in R version 3.5.2 and R studio 1.1.383 (R package ‘*lcmm*’, version 1.7.8) (Proust-Lima, Philipps, & Liquet, 2017). Notably, these trajectories should not be reified, as these are merely estimated latent groups, not actual observed groups. An advantage of latent class trajectory modeling using full information maximum likelihood estimation as applied in this study is that it allows the number of times a participant was assessed to vary between participants, which facilitates the inclusion of participants with intermittent missing data or those who dropped out (Proust-Lima et al., 2017). We used the GRoLTS guidelines and Lennon's et al. framework as a guidance to construct and interpret latent class trajectory modeling (Lennon et al., 2018; Van De Schoot et al., 2017).

Latent classes with different growth trajectories were modeled based on growth models that define how an outcome changes as a function of time, using an intercept and slope parameter. In order to find the model that best described the data, we fitted latent class growth models with fixed class-specific intercepts and slopes (LCGA), as well as more flexible growth mixture models (GMM) with (i) a random class-specific intercept and fixed slope per class and (ii) random class-specific intercepts and slopes. We fitted models with both linear and quadratic trajectories. LCGA and GMM models were fitted to the data with increasing number of classes (*g* = 1 to *g* = 7), after which indices of model fit were compared. Every model was run with multiple (25) random start values (derived from the one-class model) in order to identify a replicable Log-Likelihood maximum, that was unlikely to be at a local maximum. The best-fitting model was then fully fitted with a maximum of 500 iterations. In the models, the intercept and slope variances were constrained to be equal across classes. Data were rearranged as a function of chronological months since inclusion into the study. This resulted in 123 (baseline measurement being 0 months, the latest measurement being 123 months) instead of four assessment points, allowing for more complex trajectories to be modeled. Data points at which no valid information on the SCL-90 SOM was provided were excluded (*N* = 39 (0.02%), *N* = 767 (0.62%), *N* = 309 (0.33%), and *N* = 320 (0.36%) participants at the first, second, third, and fourth measurement, respectively). Ultimately, participants were allocated to a class based on their highest posterior class probability score. Participants with low posterior probabilities for all classes (<0.50) were excluded from the analyses.

The models with increasing number of classes were compared on four *a priori* formulated criteria: (i) the model with the lowest Bayesian Information Criterion (BIC) value was favorable (Nylund, Asparouhov, & Muthén, 2007; Van De Schoot et al., 2017); (ii) the entropy of the model with the lowest BIC was assessed, as high entropy (>0.80) indicates strong distinctive capabilities between trajectory classes (Celeux & Soromenho, 1996); (iii) class sizes, as class sizes should not comprise less than 1% of the sample (Infurna & Grimm, 2017); and (iv) theoretical plausibility, for example, verifying whether the observed trajectories fit the longitudinal plots of raw data and previous empirical findings (Ram & Grimm, 2009).

To identify the predictors of somatic symptom trajectories, we conducted multiple logistic regression analyses, including all predictors as mentioned in Table 1 as independent variables. To study whether the predictors for the latent subgroups differed between females and males, we included interaction terms between sex and predictors.

To test whether the continuous covariates included in the multiple logistic regression analyses fulfilled the linearity assumption of multiple logistic regression, we divided the covariates into quartiles, and assessed whether the estimates increased or decreased monotonically. IBM SPSS v. 25 was used to perform regression analyses. We maintained a two-sided *α*-value, corrected for multiple comparisons, of 0.001 (0.05/47, 24 predictors and 23 sex-by-predictor interaction terms within a family of tests).

Three sensitivity analyses were performed. First, we performed the regression analyses without adjusting for the presence of chronic diseases to explore its influence on the association between predictors and the identified trajectories. Second, we assessed whether the association between negative life events and the observed trajectories was partly explained by health-related negative life events. We excluded any health-related negative life events from the regression analyses to assess its influence on the association between negative life events and the identified trajectories. Third, we performed regression analyses with different symptom trajectories as a reference category.

## Results

We found that the mean SCL-90 SOM score in the complete sample remained stable over time with scores of 1.36 (s.d. = 0.38), 1.38 (s.d. = 0.45), 1.42 (s.d. = 0.44), and 1.36 (s.d. = 0.42) at subsequent measurement waves.

### Trajectory modeling

**Fig. 1.**
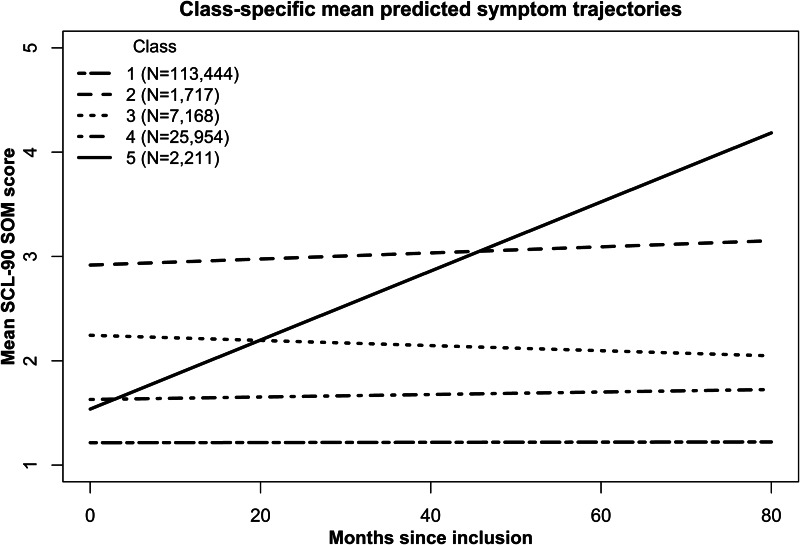
Class-specific mean predicted symptom trajectories.

Of the fitted models, LCGA performed best (online Supplementary Appendix C). Therefore, only estimates of LCGA models are shown in Table 2. The five-class model fitted the data best, as is indicated by the lowest BIC value, good entropy, and acceptable class sizes. The class-specific predicted mean SCL-90 SOM trajectories are displayed in Fig. 1. The first class, which comprises the majority of the population (*N* = 113 444; 75.4%) reported minimal to no SCL-90 SOM symptoms over time. The second class (*N* = 1717; 1.1%) reported a high, stable SCL-90 SOM symptom score over time. The third class (*N* = 7168; 4.8%) showed slightly decreasing, intermediate SCL-90 SOM symptom score, whereas the fourth class (*N* = 25 954; 17.3%) showed a low, stable SCL-90 SOM symptom score, albeit somewhat higher than the first class. The fifth class (*N* = 2211; 1.5%) started with a relatively low SCL-90 SOM score, which steeply increased over time. Online Supplementary Appendix D shows plots with individual SCL-90 SOM score trajectories, stratified per class.

**Table 2.** Parameter estimates for 1–7 classes (*N* = 150 494) using a linear trajectory function

G	NPM	Log-Likelihood	BIC^a^ value	Entropy	Participants per class (%)						Mean posterior class probability score
1	3	−254 288	508 611	100%	150 494 (100%)						1
2	6	−181 449	362 969	91.6%	132 523 (88.1%)	17 971 (11.9%)					0.98/0.92
3	9	−161 083	322 273	87.6%	24 711 (16.4%)	5516 (6.7%)	120 267 (79.9%)				0.86/0.92/0.96
4	12	−154 669	309 482	85.6%	1985 (1.3%)	8054 (5.4%)	114 618 (76.2%)	25 837 (17.2%)			0.91/0.86/0.94/0.81
5^b^	15	−149 292	298 764	86.1%	113 444 (75.4%)	1717 (1.1%)	7168 (4.8%)	25 954 (17.3%)	2211 (1.5%)		0.94/0.90/0.85/0.81/0.82
6	18	−154 669	309 553	51.8%	111 525 (74.1%)	8054 (5.4%)	28 930 (19.2%)	1985 (1.3%)	0 (0%)	0 (0%)	0.61/0.86/0.77/0.91/0/0
7	21	n.a.	n.a.	n.a.	n.a.	n.a.	n.a.	n.a.	n.a.	n.a.	Failed to converge

a Bayesian Information Criteria.

b Preferred model.

### Logistic regression analyses

Participants were allocated to one of the trajectory classes, based on their posterior class probability score; 1495 (1.0%) participants with low posterior probabilities for all classes (<0.50) were excluded from the analyses.

First, we identified the predictors of high, stable symptom severity (class 2) compared with low, stable symptom severity (class 4) by multiple logistic regression analyses (Table 3). Class 4 was selected as the reference category to facilitate a comparison with the subsequent analyses. We found that female sex was significantly associated with high symptom severity (OR 1.74, 95% CI 1.36–2.22), while femininity was associated with low symptom severity (OR 0.60, 95% CI 0.44–0.82). Also, increased physical functioning and emotional wellbeing were associated with low symptom severity (OR 0.96, 95% CI 0.96–0.97 for both predictors). On the other hand, better self-rated health was associated with high symptom severity (OR 1.03, 95% CI 1.02–1.04). We found that personality traits were not statistically significantly associated with high symptom severity. We assessed the statistical significance of the interaction term between sex and all predictors. Only the interaction terms between sex and hours of paid employment (OR 0.98, 95% CI 0.98–0.99), and sex and physical functioning (OR 0.99, 95% CI 0.98–0.99) were statistically significant, indicating that the negative association between these predictors and a high symptom severity was stronger in females than in males.

**Table 3.** The associations between predictors and high, stable symptom severity over time

		Odds ratio (95% CI)^a^		
Predictors		Total (*N* = 27 183)	Males (*N* = 8084)	Females (*N* = 19 099)
Sex	Male	1	n.a.	n.a.
	Female	1.74 (1.36–2.22)*	n.a.	n.a.
Femininity		0.60 (0.44–0.82)*	0.65 (0.39–1.08)	0.56 (0.37–0.85)*
Age		0.99 (0.98–1.00)	0.98 (0.97–1.00)	0.99 (0.98–1.00)
Education	Low	1	1	1
	Medium	0.75 (0.65–0.87)*	0.81 (0.61–1.08)	0.74 (0.62–0.88)*
	High	0.46 (0.37–0.57)*	0.43 (0.28–0.67)*	0.49 (0.38–0.63)*
Hours of paid employment^b^		0.99 (0.99–1.00)	1.00 (1.00–1.01)	0.99 (0.98–0.99)*
Presence of chronic disease		1.55 (1.33–1.81)*	1.74 (1.30–2.32)*	1.47 (1.22–1.77)*
Physical functioning^b^		0.96 (0.96–0.97)*	0.97 (0.96–0.97)*	0.96 (0.95–0.96)*
Emotional wellbeing		0.96 (0.96–0.97)*	0.96 (0.95–0.97)*	0.96 (0.96–0.97)*
Self-rated health		1.03 (1.02–1.04)*	1.04 (1.03–1.05)*	1.03 (1.03–1.04)*
Negative affect		2.20 (1.87–2.58)*	2.12 (1.56–2.86)*	2.21 (1.83–2.66)*
Positive affect		1.34 (1.14–1.57)*	1.10 (0.81–1.49)	1.35 (1.03–1.77)*
NEO PI R	Anger	1.14 (0.99–1.32)	0.91 (0.63–1.30)	1.16 (0.98–1.38)
	Self-consciousness	0.81 (0.70–0.92)*	1.14 (0.87–1.49)	0.79 (0.68–0.93)*
	Impulsivity	0.92 (0.78–1.08)	1.15 (0.81–1.62)	0.87 (0.73–1.05)
	Vulnerability	0.75 (0.63–0.90)*	0.61 (0.43–0.89)*	0.81 (0.66–1.00)
	Self-discipline	1.12 (0.96–1.30)	1.16 (0.87–1.56)	1.11 (0.93–1.33)
	Competence	0.91 (0.73–1.13)	0.99 (0.63–1.53)	0.88 (0.68–1.14)
	Deliberation	0.87 (0.75–1.01)	0.84 (0.62–1.13)	0.88 (0.74–1.06)
	Excitement	0.97 (0.85–1.10)	0.96 (0.74–1.25)	0.96 (0.82–1.12)
Occurrence of negative life event		1.17 (0.98–1.39)	1.39 (0.97–1.79)	1.11 (0.91–1.36)
Occurrence of long-term difficulty		1.23 (0.88–1.70)	0.86 (0.46–1.62)	1.38 (0.94–2.02)
Presence of mood disorder		1.05 (0.90–1.23)	1.13 (0.82–1.55)	1.03 (0.86–1.23)
Presence of anxiety disorder		1.27 (1.06–1.52)*	1.01 (0.69–1.47)	1.36 (1.11–1.67)*

a Please note that the odds presented are per unit change on the scale of the predictor, thus magnitudes are not always directly comparable. *Indicates statistical significance (*p* < 0.001). Note: Nagelkerke's *R*^2^ for the model including all participants, only the men, and only the women allocated to class 2 and class 4 are 0.38, 0.36, and 0.39, respectively.

b Interaction terms between these predictors and sex were statistically significant.

Second, we assessed which predictors were associated with increasing symptom severity, by multiple logistic regression analyses predicting increasing SCL-90 SOM score over time (class 5) versus low, stable SCL-90 SOM score (class 4), as these trajectories had a similar intercept. This allows for identification of predictors that may associate with an increasing symptom severity, instead of a low, stable symptom severity. Table 4 shows that females have 1.15 times the odds (95% CI 0.99–1.40) compared to males of having increasing symptom severity over time, however this result did not reach statistical significance. Femininity seemed to be protective of increasing symptom severity over time (OR 0.66, 95% CI 0.51–0.85), yet experiencing a negative life event is disadvantageous (OR 1.30, 95% CI 1.16–1.47). The OR of the interaction term between sex and education (OR 1.23, 95% CI 1.01–1.58), sex and the presence of chronic disease (OR 0.79, 95% CI 0.63–0.98), sex and physical functioning (OR 0.99, 95% CI 0.98–0.99), and sex and the score on the NEO-PI-R deliberation subscale (OR 1.35, 95% CI 1.10–1.64) were statistically significant, indicating that the association between these predictors and increasing symptom severity differed between females and males. The results of the sensitivity analyses, which assess the effect of the presence of chronic diseases and health-related negative life events on the association between femininity or sex and symptom trajectories, as well as the effect of selecting class 1 (no, stable symptoms) as the reference symptom trajectory instead of class 4 (low, stable symptoms) yielded essentially the same results and are shown in online Supplementary Appendix E.

**Table 4.** The associations between multiple predictors and increasing symptom severity over time

		Odds ratio (95% CI)^a^		
Predictors		Total (*N* = 27 572)	Males (*N* = 8288)	Females (*N* = 19 284)
Sex	Male	1	n.a.	n.a.
	Female	1.15 (0.99–1.40)	n.a.	n.a.
Femininity		0.66 (0.51–0.85)*	0.83 (0.55–1.24)	0.61 (0.44–0.85)*
Age		1.00 (1.00–1.01)	1.01 (1.00–1.02)	1.00 (0.99–1.01)
Education	Low	1	1	1
	Medium^b^	0.87 (0.77–0.98)*	0.71 (0.57–0.88)*	0.95 (0.83–1.01)
	High	0.71 (0.61–0.82)*	0.62 (0.47–0.80)*	0.77 (0.64–0.93)*
Hours of paid employment		1.00 (0.99–1.00)	1.00 (0.99–1.01)	0.99 (0.99–1.00)
Presence of chronic disease^b^		0.98 (0.88–1.09)	1.18 (1.01–1.42)*	0.89 (0.79–1.02)
Physical functioning^b^		1.00 (1.00–1.01)	1.01 (1.00–1.01)	1.00 (0.99–1.00)
Emotional wellbeing		1.01 (1.01–1.02)*	1.01 (1.00–1.02)	1.01 (1.00–1.02)
Self-rated health		1.00 (1.00–1.01)	1.00 (0.99–1.01)	1.00 (1.00–1.01)
Negative affect		1.12 (0.99–1.28)	0.98 (0.77–1.25)	1.18 (1.01–1.39)*
Positive affect		1.00 (0.87–1.15)	0.83 (0.65–1.06)	1.09 (0.92–1.30)
NEO PI R	Anger	1.12 (1.00–1.26)	1.23 (1.01–1.51)*	1.07 (0.93–1.24)
	Self-consciousness	1.01 (0.90–1.12)	0.99 (0.80–1.23)	1.01 (0.88–1.16)
	Impulsivity	1.03 (0.91–1.18)	1.13 (0.88–1.44)	1.00 (0.86–1.17)
	Vulnerability	0.96 (0.83–1.12)	0.88 (0.66–1.17)	0.99 (0.82–1.18)
	Self-discipline	1.17 (1.03–1.33)*	1.26 (1.00–1.58)	1.12 (0.96–1.29)
	Competence	0.89 (0.74–1.07)	0.85 (0.61–1.18)	0.90 (0.72–1.12)
	Deliberation^b^	1.02 (0.90–1.16)	0.83 (0.67–1.04)	1.11 (0.95–1.29)
	Excitement	0.97 (0.88–1.08)	1.05 (0.87–1.27)	0.94 (0.83–1.07)
Occurrence of negative life event		1.30 (1.16–1.47)*	1.11 (0.91–1.37)	1.40 (1.21–1.62)*
Occurrence of long-term difficulty		0.96 (0.81–1.13)	0.96 (0.71–1.28)	0.97 (0.79–1.18)
Presence of mood disorder		1.11 (0.99–1.31)	1.23 (0.93–1.62)	1.11 (0.94–1.30)
Presence of anxiety disorder		1.05 (0.88–1.26)	1.46 (1.04–2.04)*	0.95 (0.77–1.18)

a Please note that the odds presented are per unit change on the scale of the predictor, thus magnitudes are not always directly comparable. *Indicates statistical significance (*p* < 0.001). Nagelkerke's *R*^2^ for the model including all participants, only the men, and only the women allocated to class 5 and class 4 are 0.11, 0.28, and 0.11, respectively.

b Interaction terms between these predictors and sex were statistically significant.

## Discussion

To the best of our knowledge, this is the first large general population cohort study that assesses longitudinal somatic symptom trajectories by means of LCGA. This data-driven method allows for the identification of homogeneous patterns of symptom severity over time from heterogeneous data. We found that a five-class linear model that excluded intraclass individual variation fitted the data best. The majority of the cohort had a stable symptom trajectory (93.7%), with low (class 1; 75.4%), slightly higher (class 4; 17.3%), and high (class 2; 1.1%) symptom severity. In addition, we identified a class with slightly decreasing (class 3; 4.8%) and a class with increasing (class 5; 1.5%) symptom severity over time. We found that female sex is a predictor for a high, stable SCL-90 SOM score. However, female sex only approached statistical significance for an increasing SCL-90 SOM score, compared to male sex. Femininity, in contrast, appeared to be protective for both a stable and an increasing somatic symptom severity. In females, hours of paid employment and physical functioning were more strongly negatively associated with stable symptom severity than in males. Regarding increasing symptom severity over time, education, the presence of chronic disease, physical functioning and the score on the NEO-PI-R deliberation subscale differed in predictive strength between females and males.

### Strengths and limitations

The principal strength of this study is the data-driven approach we used to estimate common somatic symptom trajectories in a large general population cohort. Latent class trajectory modeling allows for identifying nuances between seemingly similar subpopulations (Lennon et al., 2018). This inductive approach facilitates the identification of novel predictors of at-risk subpopulations, especially if individuals' symptom trajectories are analyzed as an outcome, rather than as an independent variable. However, note that when analysis involves latent class trajectories either as outcome or exposure, one should not view the trajectories as concrete entities, but rather as a method to reduce the observed heterogeneity in the data (van Loo, Wanders, Wardenaar, & Fried, 2018). Furthermore, our cohort had a large sample size and was followed up for a long period of time, with multiple measurements, allowing for complex models to be fitted. Lastly, the incorporation of participants' gender is advantageous, as it allows for disentangling the biological and psychosocial influences related to being a woman or man on somatic symptom trajectories.

Our study had several limitations. First, we assessed the mean SCL-90 SOM score as an aggregate score and therefore we have not differentiated between individual symptoms. Possibly, participants had different symptoms that bothered them over time, despite their symptom scores remaining stable. Second, we could not account for illness cognitions or health care utilization, as no data hereon were available. Illness cognitions are thought to account for 30–40% of the variance in health outcomes related to somatic symptoms (McAndrew et al., 2019), such as the persistence of symptoms (Moss-Morris, Spence, & Hou, 2011). Similarly, health care utilization is known to associate somatic symptom burden, and thus may affect symptom trajectories (Lee, Creed, Ma, & Leung, 2015). Also, we only assessed predictors at baseline, but predictors may also have an influence during the course of one's symptoms, such as the development of chronic diseases or health care utilization. All predictors are self-reported, which means that the measures of the life-time prevalence of mood and anxiety disorders are not necessarily a clinical diagnosis, and the latter two may be more reflective of experienced mood and anxiety symptoms than of a clinical diagnosis.

### Latent class trajectories

We identified three stable symptom severity trajectories (93.7%) and two relatively small classes that follow a consistently increasing (1.5%) and decreasing (4.8%) course. The proportion of participants with non-stable trajectories is in line with previous research that used latent class trajectory modeling. In a patient population with medically unexplained somatic symptoms, 92.6% of the patients had a stable symptom score over the 2 years follow-up. The remaining 7.4% of patients improved. However, this study used the Patient Health Questionnaire-15 to measure symptom severity, which differs from the current SCL-90 SOM subscale (Claassen-van Dessel et al., 2018). Another study conducted in a general adolescent cohort showed that 85.3% of adolescents had a predominantly stable symptom trajectory over time (Janssens et al., 2014). Four trajectories were identified in this study. Again, this study used a different questionnaire, but more importantly, an adolescent cohort might not be directly comparable to an adult cohort. It is disputed whether somatic symptoms in adolescents and adults are comparable in onset, healthcare-seeking behavior, and treatment, possibly due to stronger family influences in adolescents and a differing physiology compared to adults (Weisblatt, Hindley, & Rask, 2011). Furthermore, adolescence is thought to be accompanied by a heightened bodily awareness and therefore with the experience of common somatic symptoms (Rhee, 2005). Overall, stable somatic symptom scores over time prevail in the aforementioned studies despite the differing study populations.

### Sex and gender in relation to somatic symptom trajectories

In line with our current study, recent studies have found female sex to be associated with more numerous and more severe somatic symptoms (Ballering et al., 2020; Tomenson et al., 2013), as well as with an increasing severity of somatic symptoms over time (De Gucht et al., 2004; Janssens et al., 2014). Multiple explanations have been raised for this phenomenon. First, females may have a heightened pain sensitivity due to biological differences (Fillingim, 2017). Sex hormones, genotypes, immune systems, and neurology may induce differences in the processing of pain that predispose females to worse symptom trajectories than males (Bartley & Fillingim, 2013). Second, females are thought to be more aware of bodily sensations than males. This heightened awareness allows for easier and earlier perception of somatic symptoms in females than in males (Barsky, Peekna, & Borus, 2001). These biological differences may explain the female preponderance in somatic symptoms as found in our study, but our results also point toward a role for psychosocial gender differences.

Femininity was found to be protective against both a high and increasing symptom severity. This is different from earlier cross-sectional studies that showed an association between femininity, measured by the gender index, domestic responsibilities, or the BEM sex role inventory, respectively, and higher levels of common somatic symptoms (Ballering et al., 2020; Krantz & Ostergren, 2001) or that found no association (Castro, Carbonell, & Anestis, 2012). These differences may be explained by the longitudinal nature of the current study, which provides insight into the dynamics of symptoms over time and may result in a more precise assessment of somatic symptom severity. Furthermore, in the former study in which the gender index was used, all adult Lifelines participants were included and femininity was found to be associated with more severe symptoms. However, the current study focusses on participants with high or increasing symptom severity (1.1% and 1.5% of the adult participants, respectively) and femininity was not found to associate with increased symptom severity. Possibly increased healthcare-seeking behavior plays a role in femininity being a protective factor for both high, stable and increasing symptom severity over time (Steinbrecher & Hiller, 2011), as healthcare-seeking behavior is known to be gendered (Hart, Saperstein, Magliozzi, & Westbrook, 2019) and may prevent worsening of symptoms over time (Lee et al., 2015). Feminine people are thought to have a lower threshold to seek help or medical care, especially from their GP (Loikas et al., 2015). Femininity is, for example, related to providing and facilitating care for the family, allowing feminine people to be more often in contact with healthcare providers, concomitantly lowering the barrier for healthcare-seeking behavior. Additionally, femininity is related to being open and less stoic about one's symptoms, facilitating healthcare-seeking behavior. Masculinity, in contrast, relates to being less expressive about distress and seeking help for symptoms is stereotypically seen as socially undermining an individual's masculinity (Barsky et al., 2001; MacLean, Sweeting, & Hunt, 2010). The earlier study that used the gender index included participants with low symptom severity as well, in which healthcare-seeking behavior may not be as important.

In addition, what constitutes femininity differs between studies and changes over time and place, yielding different results. For example, the BEM sex role inventory was developed in 1974 and then widely applied, but is currently deemed to hold limited validity as an operationalization of femininity or masculinity (Donnelly & Twenge, 2017). Lastly, the association between femininity and higher levels of somatic symptom severity in the previous studies may have been partially explained by the presence of chronic diseases, whilst we adjusted for this in the current study. Sensitivity analyses showed that indeed an adjustment for the presence of chronic diseases slightly strengthened the protective association between femininity and high or increasing symptom severity over time.

### Predictors of stable and increasing common somatic symptoms

In addition to sex and gender, multiple factors were predictive of persistent common somatic symptoms. Higher education, higher levels of physical functioning, and higher emotional wellbeing at baseline are associated with low, stable symptom severity. This is in line with previous research that suggests that these factors have a positive influence on overall functioning and wellbeing (van Eck van der Sluijs et al., 2018). Here, we also found that the lower one rates his or her own general health, the lower the odds that one has persisting common somatic symptoms. Perceptions of low general health may prompt one to seek medical help, which may lead to an improvement of symptoms (Steinbrecher & Hiller, 2011). However, previous studies contradict each other with regards to self-rated health and the course of somatic symptoms (Janssens et al., 2014; Steinbrecher & Hiller, 2011; van Eck van der Sluijs et al., 2018). These contradictions might be due to the different conceptualizations of self-related health and somatic symptoms in the studies.

We also found that the presence of anxiety disorders is related to stable and increasing symptom severity in females. Anxiety disorders are diagnosed approximately twice as often in females than in males (McLean, Asnaani, Litz, & Hofmann, 2011), and often manifest themselves with prominent somatic characteristics (Bekhuis, Schoevers, Van Borkulo, Rosmalen, & Boschloo, 2016). Therefore, the presence of anxiety disorders may contribute to the elevated SCL-90 SOM scores as found in this study. A self-reported mood disorder, however, was not associated with a high or increasing symptom severity. Evidence from cross-sectional studies suggests that mood disorders are associated with more severe common somatic symptoms (Bekhuis, Boschloo, Rosmalen, & Schoevers, 2015; Löwe et al., 2008). In contrast, results of longitudinal research assessing mood disorders in relation to somatic symptoms are contradictive (Niles & O'Donovan, 2019; Steinbrecher & Hiller, 2011; van Eck van der Sluijs et al., 2018). To date, it has not been possible to draw any definitive conclusion on whether mood disorders predict an unfavorable somatic symptom prognosis. It has been argued that a similar mechanism as mentioned above may apply to people with mood disorders: being affected by mood disorders may prompt healthcare-seeking behavior, resulting in an improvement of symptoms. The aforementioned longitudinal studies, however, including our study, do not differentiate between, or assess different, somatic symptoms. Thus the association between mood disorders and one type of symptom may be overshadowed by the lack of an association with other types of symptoms.

We also found that negative life events are predictors of increasing symptom severity. As a sensitivity analysis, we removed any item from the negative life events scale that was related to experiencing a severe disease . The direction and strength of the association remained similar. It is thought that psychological distress as a consequence of a negative life event may result in somatic symptoms (Tak, Kingma, van Ockenburg, Ormel, & Rosmalen, 2015). Physiological and emotional stress mechanisms are suggested as the link between psychological distress and somatic symptoms (Bonvanie, Janssens, Rosmalen, & Oldehinkel, 2017). Such mechanisms heighten one's bodily vigilance, consequently facilitating people to interpret bodily signals more easily as somatic symptoms.

### Implications for further research and clinical practice

Further research could focus on symptom-specific latent class trajectories, to assess whether differences in trajectories exist between symptoms. Additionally, one could study whether the type of reported symptoms change over time in the classes with stable, high mean symptom severity scores and whether these changes follow specific sequences. The results from this study also show that the majority of the general population remains stable in their level of symptom severity and that only a relatively small proportion has a high or increasing symptom severity. However, it remains unknown whether it is merely the latter population that seeks medical attention and if so, what factors are associated with this healthcare-seeking behavior. As a protective association between femininity and a high and increasing symptom severity was found in this study, it is especially interesting to study to what extent femininity relates to healthcare-seeking behavior for somatic symptoms. For those patients who visit their GP, it is pivotal that predictors for increasing symptom severity are recognized, preferably in an early stage.

We found no large sex differences in the predictors of high or increasing symptom severity, thus it may not be clinically useful to distinguish between predictors specific to male or female patients withpersistent common somatic symptoms. Furthermore, for reasons of clarity, we currently described the associations of sex and gender with common somatic symptom trajectories separately. However, although sex and gender are different concepts, a clear demarcation between these in clinical practice is artificial: clinicians cannot consider sex without gender and *vice versa* as these concepts are intertwined.

## Supplementary material

For supplementary material accompanying this paper visit https://doi.org/10.1017/S0033291720004043. click here to view supplementary material
