# Supplementary material for: Female sex and femininity independently associate with common somatic symptom trajectories
Source: Psychol Med. 2020 Nov 10;52(11):2144–54. doi: 10.1017/S0033291720004043 (PMC9386437; doi:10.1017/S0033291720004043)
Supplement: Supplementary file 1 [file S0033291720004043sup001.docx]

**Appendix A: Demographic characteristics of the study population at baseline (N=150,494).**

|  | | | **Men** (N=62,364; 41.4%) | **Women** (N=88,130; 58.6%) |
| --- | --- | --- | --- | --- |
| **Mean age in years (SD)** | | | 45.1 (13.0) | 44.0 (12.9) |
| **Education** | **Low** | | 19,432 (31.2%) | 27,869 (31.6%) |
|  | **Medium** | | 23,562 (37.8%) | 35,166 (39.9%) |
|  | **High** | | 19,370 (31.1%) | 25,095 (28.5%) |
| **Median gender index (IQR)** | | | 0.06 (0.01-0.24) | 0.96 (0.83-0.99) |
| **Median SCL-90 SOM sumscore** | | | 14.0 (13.0-17.0) | 14.0 (13.0-17.0) |
| **Presence of chronic physical disease** | | **No** | 38,404 (61.6%) | 45,000 (51.1%) |
|  | | **Yes** | 23,960 (38.4%) | 43,130 (48.9%) |
| **Employment** | | **No** | 10,244 (16.6%) | 19,774 (22.7%) |
|  | | **Yes** | 51,343 (83.4%) | 67,223 (77.3%) |
| **Experienced a longterm difficulty** | | **No** | 15,047 (25.2%) | 17,369 (20.6%) |
|  | | **Yes** | 44,736 (74.8%) | 66,928 (79.4%) |
| **Experienced a negative life event** | | **No** | 25,101 (42.0%) | 31,808 (37.7%) |
|  | | **Yes** | 34,693 (58.0%) | 52,513 (62.3%) |

**Appendix B: The SCL-90 SOM subscale**The Symptom CheckList-90 somatization subscale (SCL-90 SOM) is part of the Symptom CheckList-90 (SCL-90). It asks participants to score how much they were bothered or distressed by twelve somatic symptoms in the past week. The total score may range from 12–60, whereas the mean score of the SCL-90 SOM may range from 1-5.

| **How much in the past week were you bothered by:** | | **Not at all** | **A little bit** | **Moderately** | **Quite a bit** | **Extremely** |
| --- | --- | --- | --- | --- | --- | --- |
| **1** | Headache | 1 | 2 | 3 | 4 | 5 |
| **2** | Dizziness | 1 | 2 | 3 | 4 | 5 |
| **3** | Chest pain | 1 | 2 | 3 | 4 | 5 |
| **4** | Lower back pain | 1 | 2 | 3 | 4 | 5 |
| **5** | Nausea | 1 | 2 | 3 | 4 | 5 |
| **6** | Painful muscles | 1 | 2 | 3 | 4 | 5 |
| **7** | Difficulties breathing | 1 | 2 | 3 | 4 | 5 |
| **8** | Feeling hot and cold alternately | 1 | 2 | 3 | 4 | 5 |
| **9** | Numbness or tingling in parts of your body | 1 | 2 | 3 | 4 | 5 |
| **10** | Feeling a lump in your throat | 1 | 2 | 3 | 4 | 5 |
| **11** | Weakness in body parts | 1 | 2 | 3 | 4 | 5 |
| **12** | Heavy arms or legs | 1 | 2 | 3 | 4 | 5 |

**Appendix C: Overview of the fit indices of the fitted models**

| **G** | **Polynomial degree** | **NPM** | **Random effects** | **Log-Like** | **BIC** | **Entropy** | **Participants per class (%)** | | | | | | **Mean class membership posterior probabilities** |
| --- | --- | --- | --- | --- | --- | --- | --- | --- | --- | --- | --- | --- | --- |
| **1** | Linear | 3 | 0 | -254,288 | 508,611 | 100% | 150,494 (100%) | | | | | | 1 |
|  | Linear | 4 | 1 | -190,022 | 380,092 | 100% | 150,494 (100%) | | | | | | 1 |
|  | Linear | 6 | 2 | -189,296 | 378,664 | 100% | 150,494 (100%) | | | | | | 1 |
|  |  |  |  |  |  |  |  | | | | | |  |
| **2** | Linear | 6 | 0 | -181,449 | 362,969 | 91.6% | 132,523  (88.1%) | 1,7971  (11.9%) | | | | | 0.98/0.92 |
|  | Linear | 7 | 1 | -167,150 | 334,384 | 92.3% | 138,431  (92.0%) | 12,063  (8.0%) | | | | | 0.98/0.91 |
|  | Linear | 9 | 2 | -167,051 | 334,210 | 92.4% | 138,347  (91.9%) | 12,147  (8.1%) | | | | | 0.98/0.90 |
|  |  |  |  |  |  |  |  | | | | | |  |
| **3** | Linear | 9 | 0 | -161,083 | 322,273 | 87.6% | 24,711  16.4% | 5,516  6.7% | 120,267  79.9% | | | | 0.86/0.92/0.96 |
|  | Linear | 10 | 1 | -167,150 | 334,419 | 45.2% | 137,477  (91.4%) | 13,017  (8.6%) | 0  (0%) | | | | 0.86/0.70/0 |
|  | Linear | 12 | 2 | -167,051 | 334,246 | 40.7% | 137,160  (91.1%) | 0  (0%) | 13,334  (8.9%) | | | | 0.63/0/0.86 |
|  |  |  |  |  |  |  |  | | | | | |  |
| **4** | Linear | 12 | 0 | -154,669 | 309,482 | 85.6% | 1,985  (1.3%) | 8,054  (5.4%) | 114,618  (76.2%) | 25,837  (17.2%) | | | 0.91/0.86/0.94/0.81 |
|  | Linear | 13 | 1 | -167,150 | 334,456 | 24.7% | 14,628  (9.7%) | 0  (0%) | 135,866  (90.3%) | 0  (0%) | | | 0.81/0/0.38/0 |
|  | Linear | 15 | 2 | -167,051 | 334,282 | 24.4% | 14,833  (9.9%) | 0  (0%) | 135,661  (90.1%) | 0  (0%) | | | 0.81/0/0.36/0 |
|  |  |  |  |  |  |  |  | | | | | |  |
| **5** | Linear | 15 | 0 | -149,292 | 298,764 | 86.1% | 113,444  (75.4%) | 1,717  (1.1%) | 7,168  (4.8%) | 25,954  (17.3%) | 2,211  (1.5%) | | 0.94/0.90/0.85/0.81/0.82 |
|  | Linear | 16 | 1 | -167,150 | 334,491 | 18.1% | 15,381  (10.2%) | 135,113  (89.8%) | 0  (0%) | 0  (0%) | 0  (0%) | | 0.78/0.27/0/0/0 |
|  | Linear | 18 | 2 | -167,051 | 334,317 | 18.4% | 135,050  (89.7) | 154,44  (10.4%) | 0  (0%) | 0  (0%) | 0  (0%) | | 0.27/0.78/0/0/0 |
|  |  |  |  |  |  |  |  | | | | | |  |
| **6** | Linear | 18 | 0 | -154,669 | 309,553 | 51.8% | 111,525  (74.1%) | 8,054  (5.4%) | 28,930  (19.2%) | 0  (0%) | 1,985  (1.3%) | 0  (0%) | 0.61/0.86/0.77/0/0.91/0 |
|  | Linear | 19 | 1 |  |  |  |  | | | | | | Failed to converge |
|  | Linear | 21 | 2 |  |  |  |  | | | | | | Failed to converge |
|  |  |  |  |  |  |  |  | | | | | |  |
| 7 | Linear | 21 | 0 |  |  |  |  | | | | | | Failed to converge |
|  | Linear | 22 | 1 |  |  |  |  | | | | | | Failed to converge |

|  | Linear | 24 | 2 |  |  |  |  | Failed to converge |
| --- | --- | --- | --- | --- | --- | --- | --- | --- |

Reported are: the number of latent classes; the model’s polynomial form; the number of estimated parameters; the presence of random effects in the model with 0 being no random effect (LCGA), 1 allowing for individual variance around the class’ intercept (GMM), 2 allowing for individual variance of both the intercept and slope around the class’ mean (GMM); the maximum Log-Likelihood (Log-Like); the Bayesian Information Criterion (BIC) value; the model’s entropy; and for models with g≥2 classes, the *a-posteriori* classification of participants in each class and; the mean of posterior probabilities in each latent class. To ensure that the models did not converge on local maxima of the Log-Likelihood, all models were estimated by means of a grid search. This indicates that multiple models with different randomly selected starting values are explored. Use of a grid search ensures that the estimated parameters reflect the global maximum Log-Likelihoods. The variance/covariance matrix was constrained over latent classes and non-structured.

| **G** | **Polynomial degree** | **NPM** | **Random effects** | **Log-Like** | **BIC** | **Entropy** | **Participants per class (%)** | | | | | **Mean class membership posterior probabilities** |
| --- | --- | --- | --- | --- | --- | --- | --- | --- | --- | --- | --- | --- |
| **1** | Quadratic | 4 | 0 | -254,097 | 508,243 | 100% | 150,494 (100%) | | | | | 1 |
|  | Quadratic | 5 | 1 | -189,526 | 379,111 | 100% | 150,494 (100%) | | | | | 1 |
|  | Quadratic | 10 | 2 | -187,738 | 375,595 | 100% | 150,494 (100%) | | | | | 1 |
|  |  |  |  |  |  |  |  | | | | |  |
| **2** | Quadratic | 8 | 0 | -180,602 | 361,299 | 91.5% | 132,124  (87.8%) | 18,370  (12.2%) | | | | 0.98/0.92 |
|  | Quadratic | 9 | 1 | -165,580 | 331,267 | 92.3% | 11,749  (7.8%) | 138,745  (92.2%) | | | | 0.89/0.99 |
|  | Quadratic | 14 | 2 | -187,738 | 375,643 | 0% | 95,002  (63.1%) | 55,492  (36.9%) | | | | 0.50/0.50 |
|  |  |  |  |  |  |  |  | | | | |  |
| **3** | Quadratic | 12 | 0 | -159,737 | 319,616 | 87.4% | 5,504  (3.7%) | 120,203  (79.9%) | 24,787  (16.5%) | | | 0.93/0.96/0.86 |
|  | Quadratic | 13 | 1 | -165,580 | 331,315 | 50.3% | 138,018  (92.2%) | 0  (0%) | 12,476  (8.3%) | | | 0.76/0/0.87 |
|  | Quadratic | 18 | 2 | -165,042 | 330,298 | 0% | 137,838  (91.6%) | 12,656  (8.4%) | 0  (0%) | | | 0.70/0.87/0 |
|  |  |  |  |  |  |  |  | | | | |  |
| **4** | Quadratic | 16 | 0 | -159,737 | 319,664 | 66.8% | 5,499  (3.7%) | 119,622  (79.5%) | 0  (0%) | 25,373  (16.9%) | | 0.93/0.82/0/0.85 |
|  | Quadratic | 17 | 1 | -165,580 | 331,362 | 26.5% | 137,083  (91.1%) | 13,411  (8.9%) | 0  (0%) | 0  (0%) | | 0.47/0.84/0/0 |
|  | Quadratic | 22 | 2 | -165,042 | 330,346 | 0% | 136,240  (90.5%) | 14,254  (9.5%) | 0  (0%) | 0  (0%) | | 0.37/0.81/0/0 |
|  |  |  |  |  |  |  |  | | | | |  |
| **5** | Quadratic | 20 | 0 | -153,087 | 306,412 | 78.5% | 0  (0%) | 8,121  (5.4%) | 25,704  (17.1%) | 114,499  (76.1%) | 2,170  (1.4%) | 0/0.84/0.8/0.90/0.91 |
|  | Quadratic |  | 1 |  |  |  |  |  |  |  |  | Failed to converge |
|  | Quadratic |  | 2 |  |  |  |  |  |  |  |  | Failed to converge |

**Appendix D: Individual SCL-90 SOM trajectories over time, stratified by class.**Note: for reasons of clarity, N=5,000 participants are shown per class if the class size exceeds 5,000 participants.


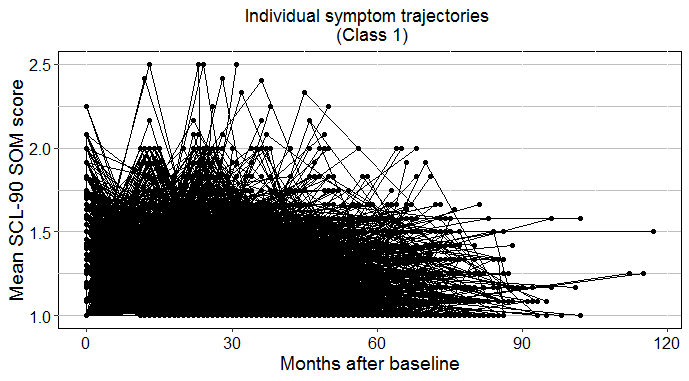

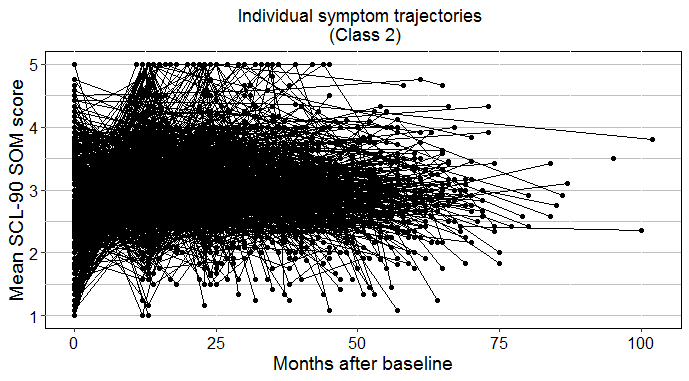

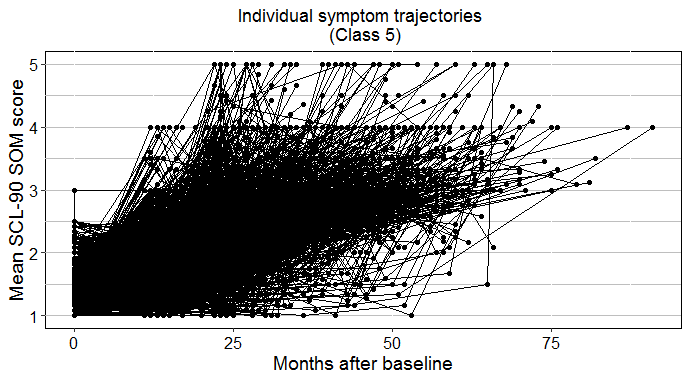

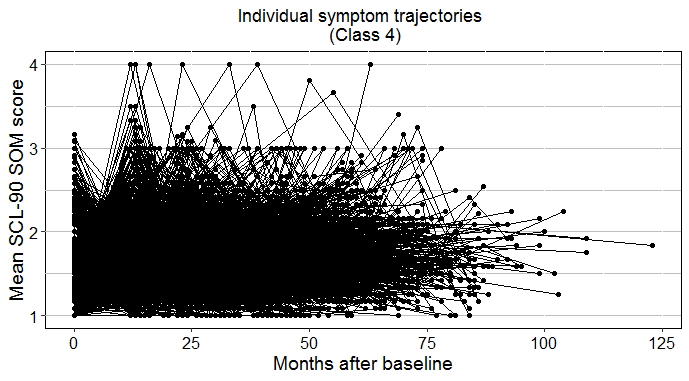

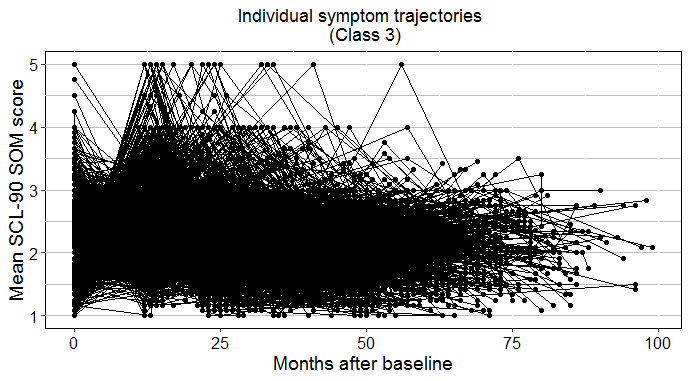


**Appendix E: Sensitivity Analyses**

| **Sensitivity analyses: exploring the effect of chronic diseases on the association between femininity and high symptom severity.** | | | | | |
| --- | --- | --- | --- | --- | --- |
|  | | | | **Odds ratio (95% CI)** | |
| **Predictors** | | | | **Total (N=27,183)** | |
| **Sex** | **Male** | | | 1 | 1 |
|  | **Female** | | | 1.36 (1.11-1.66) | 1.31 (1.07-1.60) |
| **Femininity** | | | | 0.92 (0.73-1.16) | 0.84 (0.64-1.07) |
| **Age** | | | | 1.00 (1.00-1.01) | 1.00 (0.99-1.00) |
| **Education** | **Low** | | | 1 | 1 |
|  | **Medium** | | | 0.52 (0.47-0.58) | 0.52 (0.47-0.58) |
|  | **High** | | | 0.28 (0.23-0.33) | 0.28 (0.24-0.33) |
| **Presence of chronic disease** | | | | n.a. | 2.37 (2.10-2.67) |
| **Sensitivity analyses: the effect of chronic diseases on the association between femininity and increasing symptom severity.** | | | | | |
|  | |  |  | **Odds ratio (95% CI)** | |
| **Predictors** | |  |  | **Total (N=27,572)** | |
| **Sex** | | **Male** | | 1 | 1 |
|  | | **Female** | | 1.30 (1.09-1.55) | 1.30 (1.09-1.56) |
| **Femininity** | | | | 0.70 (0.57-0.87) | 0.67 (0.54-0.84) |
| **Age** | | | | 1.02 (1.02-1.03) | 1.02 (1.02-1.03) |
| **Education** | | **Low** | | 1 | 1 |
|  | | **Medium** | | 0.82 (0.74-0.91) | 0.82 (0.74-0.92) |
|  | | **High** | | 0.66 (0.58-0.75) | 0.65 (0.58-0.74) |
| **Presence of chronic disease** | | | | n.a. | 0.94 (0.86-1.04) |

| **Sensitivity analyses: exploring the effect of any health-related negative life event on the association between negative life events and high symptom severity.** | | | | |
| --- | --- | --- | --- | --- |
|  | | | **Odds ratio (95% CI)** | |
| **Predictors** | | | **Total (N=27,183)** | |
| **Sex** | **Male** | | 1 | 1 |
|  | **Female** | | 1.35 (1.10-1.67) |  |
| **Femininity** | | | 0.84 (0.66-1.08) | 0.84 (0.66-1.08) |
| **Age** | | | 1.00 (0.99-1.00) | 1.00 (0.99-1.00) |
| **Education** | **Low** | | 1 | 1 |
|  | **Medium** | | 0.54 (0.49-0.62) | 0.55 (0.49-0.62) |
|  | **High** | | 0.30 (0.25-0.36) | 0.30 (0.25-0.36) |
| **Presence of chronic disease** | | | 2.53 (2.22-2.88) | 2.54 (2.23-2.90) |
| **Occurrence of negative life event** | | | 2.21 (1.92-2.55) | 2.12 (1.85-2.45)^a^ |
| **Sensitivity analyses: exploring the effect of any health-related negative life event on the association between negative life events and increasing symptom severity.** | | | | |
|  | |  | **Odds ratio (95% CI)** | |
|  | |  | **Total (N=27,572)** | |
| **Sex** | | **Male** | 1 | 1 |
|  | | **Female** | 1.32 (1.10-1.58) | 1.32 (1.10-1.58) |
| **Femininity** | | | 0.68 (0.55-0.84) | 0.68 (0.55-0.84) |
| **Age** | | | 1.02(1.02-1.02) | 1.02(1.02-1.02) |
| **Education** | | **Low** | 1 | 1 |
|  | | **Medium** | 0.82 (0.73-0.91) | 0.82 (0.73-0.91) |
|  | | **High** | 0.64 (0.56-0.73) | 0.64 (0.56-0.73) |
| **Presence of chronic disease** | | | 0.97 (0.88-1.06) | 0.97 (0.88-1.06) |
| **Occurrence of negative life event** | | | 1.23 (1.11-1.36) | 1.23 (1.11-1.36)^a^ |
| ^a^This odds ratio reflects the association between the occurrence of non-health-related negative life and high or increasing symptom severity. | | | | |

| **Sensitivity analyses: The associations between predictors and high, stable symptom severity over time (class 2), with no symptoms (class 1) as a reference group.** | | | | |
| --- | --- | --- | --- | --- |
|  | | **Odds ratio (95% CI)^a^** | | |
| **Predictors** | | **Total (N=114,381)** | **Men (N=51,724)** | **Women (N=62,657)** |
| **Sex** | **Male** | 1 | n.a. | n.a. |
|  | **Female** | **2.19 (1.67–2.86)*** | n.a. | n.a. |
| **Femininity** | | **0.68 (0.48-0.96)*** | 0.71 (0.40-1.25) | 0.64 (0.41-1.01) |
| **Age** | | 0.99 (0.98-1.00) | 0.99 (0.97-1.00) | 0.99 (0.98-1.00) |
| **Education** | **Low** | 1 | 1 | 1 |
|  | **Medium** | **0.63 (0.53-0.74)*** | **0.64 (0.47-0.88)*** | **0.63 (0.52-0.77)*** |
|  | **High** | **0.34 (0.27-0.42)*** | **0.29 (0.18-0.46)*** | **0.36 (0.28-0.47)*** |
| **Hours of paid employment** | | 0.99 (0.99-1.00) | 1.00 (0.99-1.01) | **0.99 (0.98-0.99)*** |
| **Presence of chronic disease** | | **2.15 (1.82-2.55)*** | **2.57 (1.87-3.52)*** | **2.02 (1.66-2.47)*** |
| **Physical functioning** | | **0.94 (0.94-0.95)*** | **0.94 (0.94-0.95)*** | **0.94 (0.94-0.94)*** |
| **Emotional wellbeing** | | **0.95 (0.94-0.96)*** | **0.95 (0.94-0.96)*** | **0.95 (0.94-0.96)*** |
| **Self-rated health** | | **1.06 (1.06-1.07)*** | **1.07 (1.05-1.08)*** | **1.06 (1.05-1.07)*** |
| **Negative affect** | | **2.94 (2.47-3.52)*** | **2.73 (1.95-3.82)** | **3.03 (2.46-3.73)*** |
| **Positive affect** | | **1.46 (1.21-1.76)*** | 1.23 (0.86-1.75) | **1.55 (1.24-1.94)*** |
| **NEO PI R** | **Anger** | **1.48 (1.25–1.74)*** | **1.57 (1.16-2.12)*** | **1.47 (1.21-1.78)*** |
|  | **Self-consciousness** | **0.78 (0.66–0.91)*** | 0.83 (0.60-1.14) | **0.76 (0.63-0.91)*** |
|  | **Impulsivity** | 0.96 (0.81-1.17) | 1.19 (0.81-1.73) | 0.93 (0.75-1.15) |
|  | **Vulnerability** | **0.77 (0.63-0.95)*** | **0.51 (0.34-0.76)*** | 0.89 (0.70-1.13) |
|  | **Self-discipline** | **1.20 (1.01-1.43)*** | 1.11 (0.79-1.56) | **1.25 (1.02-1.54)*** |
|  | **Competence** | 0.92 (0.72-1.19) | 1.02 (0.63-1.67) | 0.89 (0.66-1.20) |
|  | **Deliberation** | 0.96 (0.80-1.14) | 0.91 (0.65-1.28) | 0.97 (0.79-1.19) |
|  | **Excitement** | 0.96 (0.82-1.11) | 0.88 (0.67-1.17) | 0.98 (0.82-1.17) |
| **Occurrence of negative life event** | | **1.44 (1.19-1.74)*** | **2.03 (1.39-2.97)*** | **1.28 (1.03-1.59)*** |
| **Occurrence of long term difficulty** | | **1.72 (1.24-2.40)*** | 1.42 (0.75-2.68) | **1.85 (1.25-2.73)*** |
| **Presence of mood disorder** | | **1.34 (1.12-1.61)*** | **1.83 (1.27-2.65)*** | 1.22 (0.98-1.51) |
| **Presence of anxiety disorder** | | **1.52 (1.23-1.88)*** | 1.24 (0.79-1.94) | **1.64 (1.28-2.09)*** |
| ^a^Please note that the odds presented are per unit change on the scale of the predictor, thus magnitudes are not always directly comparable. *Indicates statistical significance (*p*<0.001). Nagelkerke’s R^2^ for the model including all participants, only the men and only the women allocated to class 2 and class 1 are 0.59, 0.58 and 0.59, respectively. | | | | |

| **Sensitivity analyses: The associations between multiple predictors and increasing symptom severity (class 5) over time, with no symptoms (class 1) as a reference group.** | | | | |
| --- | --- | --- | --- | --- |
|  | | **Odds ratio (95% CI)^a^** | | |
| **Predictors** | | **Total (N=114,770)** | **Men (N=51,928)** | **Women (N=64,842)** |
| **Sex** | **Male** | 1 | n.a. | n.a. |
|  | **Female** | **1.76 (1.45-2.13)*** | n.a. | n.a. |
| **Femininity** | | **0.71 (0.55-0.91)*** | 0.78 (0.52-1.16) | **0.70 (0.50-0.98)*** |
| **Age** | | 1.01 (1.00-1.01) | 1.02 (1.00-1.02) | 1.00 (0.99-1.01) |
| **Education** | **Low** | 1 | 1 | 1 |
|  | **Medium** | **0.74 (0.66-0.83)*** | **0.60 (0.49-0.74)*** | **0.82 (0.71-0.95)*** |
|  | **High** | **0.46 (0.40-0.54)*** | **0.39 (0.30-0.51)*** | **0.51 (0.42-0.62)*** |
| **Hours of paid employment** | | 1.00 (0.99-1.00) | 1.00 (0.99-1.01) | 0.99 (0.99-1.00) |
| **Presence of chronic disease** | | **1.48 (1.33-1.65)*** | **1.84 (1.53-2.22)*** | **1.34 (1.17-1.52)*** |
| **Physical functioning** | | **0.97 (0.97-0.97)*** | **0.98 (0.97-0.98)** | **0.97 (0.97-0.97)*** |
| **Emotional wellbeing** | | **0.99 (0.98-0.99)*** | **0.99 (0.98-0.99)*** | **0.99 (0.98-0.99)*** |
| **Self-rated health** | | **1.02 (1.02-1.03)*** | **1.02 (1.02-1.03)*** | **1.02 (1.02-1.03)*** |
| **Negative affect** | | **1.67 (1.47-1.90)*** | **1.49 (1.18-1.89)*** | **1.75 (1.50-2.05)*** |
| **Positive affect** | | 1.07 (0.93-1.24) | 0.81 (0.63-1.04) | **1.25 (1.05-1.48)*** |
| **NEO PI R** | **Anger** | **1.32 (1.18-1.49)*** | **1.47 (1.20-1.80)*** | **1.24 (1.07-1.43)*** |
|  | **Self-consciousness** | 0.99 (0.88-1.11) | 0.94 (0.76-1.17) | 1.00 (0.87-1.15) |
|  | **Impulsivity** | **1.25 (1.10-1.43)*** | **1.49 (1.17-1.90)*** | 1.17 (0.99-1.36) |
|  | **Vulnerability** | 0.96 (0.83-1.12) | 0.86 (0.65-1.14) | 0.97 (0.81-1.16) |
|  | **Self-discipline** | **1.21 (1.06-1.37)*** | **1.30 (1.03-1.63)*** | 1.15 (0.98-1.34) |
|  | **Competence** | 0.97 (0.80-1.17) | 0.98 (0.70-1.36) | 0.96 (0.76-1.21) |
|  | **Deliberation** | 1.08 (0.95-1.22) | 0.93 (0.74-1.16) | 1.15 (0.99-1.34) |
|  | **Excitement** | 0.96 (0.86-1.06) | 1.02 (0.84-1.22) | 0.94 (0.83-1.07) |
| **Occurrence of negative life event** | | **1.55 (1.38-1.74)*** | **1.34 (1.10-1.64)*** | **1.67 (1.44-1.93)*** |
| **Occurrence of long term difficulty** | | **1.42 (1.20-1.66)*** | **1.47 (1.11-1.94)*** | **1.39 (1.14-1.69)*** |
| **Presence of mood disorder** | | **1.44 (1.24-1.66)*** | **1.52 (1.15-2.02)*** | **1.41 (1.19-1.67)*** |
| **Presence of anxiety disorder** | | **1.28 (1.07-1.54)*** | **1.72 (1.22-2.41)*** | 1.16 (0.93-1.44) |
| ^a^Please note that the odds presented are per unit change on the scale of the predictor, thus magnitudes are not always directly comparable. *Indicates statistical significance (*p*<0.001). Nagelkerke’s R^2^ for the model including all participants, only the men and only the women allocated to class 5 and class 4 are 0.13, 0.14 and 0.12, respectively. | | | | |
